# Handshakes and Fights: The Regulatory Interplay of RNA-Binding Proteins

**DOI:** 10.3389/fmolb.2017.00067

**Published:** 2017-09-29

**Authors:** Erik Dassi

**Affiliations:** Centre for Integrative Biology, University of Trento, Trento, Italy

**Keywords:** RNA-binding proteins, post-transcriptional regulation, regulatory networks, regulatory elements, cooperation, competition, autoregulation

## Abstract

What drives the flow of signals controlling the outcome of post-transcriptional regulation of gene expression? This regulatory layer, presiding to processes ranging from splicing to mRNA stability and localization, is a key determinant of protein levels and thus cell phenotypes. RNA-binding proteins (RBPs) form a remarkable army of post-transcriptional regulators, strong of more than 1,500 genes implementing this expression fine-tuning plan and implicated in both cell physiology and pathology. RBPs can bind and control a wide array of RNA targets. This sheer amount of interactions form complex regulatory networks (PTRNs) where the action of individual RBPs cannot be easily untangled from each other. While past studies have mostly focused on the action of individual RBPs on their targets, we are now observing an increasing amount of evidence describing the occurrence of interactions between RBPs, defining how common target RNAs are regulated. This suggests that the flow of signals in PTRNs is driven by the intertwined contribution of multiple RBPs, concurrently acting on each of their targets. Understanding how RBPs cooperate and compete is thus of paramount importance to chart the wiring of PTRNs and their impact on cell phenotypes. Here we review the current knowledge about patterns of RBP interaction and attempt at describing their general principles. We also discuss future directions which should be taken to reach a comprehensive understanding of this fundamental aspect of gene expression regulation.

## Introduction

The regulatory interplay phenomenon occurs when two RNA-binding proteins (RBPs) concur to regulate a common RNA target. Their combined action thus defines the steady-state levels of this RNA, its availability for translation (mRNA) or ability to exert its role (non-coding RNA). The two interacting RBPs can either be synergistic (*cooperate*), aim for a different regulatory outcome (*compete*), or regulate one another to tune their action (*mutual control*).

RNA-binding proteins are major players in post-transcriptional control of gene expression (PTR), the regulatory layer responsible for fine-tuning protein synthesis and thus determining cell phenotypes (Gerstberger et al., 2014). PTR is involved in a wide range of processes, from splicing and alternative polyadenylation to mRNA localization, storage, and degradation, ultimately leading to translational control (Glisovic et al., 2008). The action exerted by PTR has profound implications for both cell physiology and pathologies such as cancer (Wurth and Gebauer, 2015) and neurodegenerative diseases (De Conti et al., 2017).

RBPs represent a formidable “army” of post-transcriptional regulators, including over 1,500 human genes geared at implementing the PTR expression fine-tuning plan. Their structure is highly modular, often including multiple domains dictating the binding specificity by independently making contact with the RNA (Lunde et al., 2007). RBPs control a wide array of targets, as revealed by techniques based on crosslinking and immunoprecipitation (CLIPs; Wheeler et al., 2017). These interactions, happening in the context of ribonucleoprotein complexes, give rise to complex post-transcriptional regulatory networks (PTRNs), whose output eventually determines cell phenotypes. Efforts to identify both RBP targets and the RBPs regulating specific RNAs have revealed several cases of RBP-RBP interplay under different patterns. Also, an early large-scale analysis of a few yeast RBPs suggested the pervasive occurrence of combinatorial regulation (Hogan et al., 2008).

Given the role these mechanisms can have in shaping the wiring and the output of PTRNs, it is essential to understand them and characterize their role in physiology and pathology. Here we review our current knowledge of RBP interplay patterns, discuss the various forms they take and their role in shaping post-transcriptional regulatory networks.

## Cooperative target regulation

**Figure 1:**
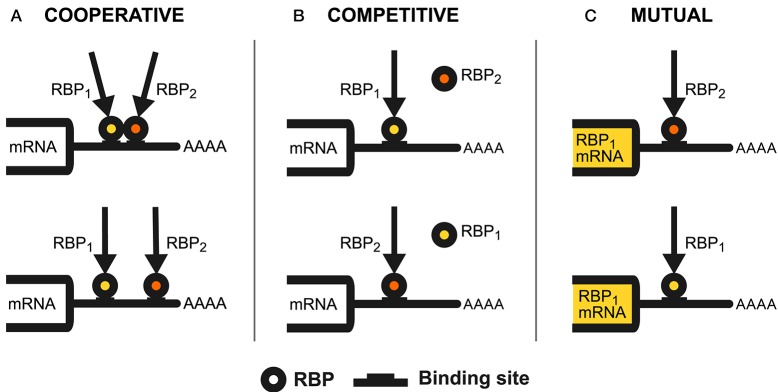
RBPs regulatory interplay modes. The figure shows the different patterns of regulatory interaction observed between RNA-binding proteins. We use a hypothetical mRNA 3′UTR as the interaction substrate to illustrate these mechanisms. **(A)** Describes the cooperative interplay mode. We have proximal cooperative binding (up) when two RBPs physically interact through nearby binding sites (or distant sites brought in proximity by the RNA secondary structure conformation), thus shaping their action through this interaction. RBPs can also cooperate through distant binding sites and without interacting directly (down), exerting their synergistic activity independently from one another. **(B)** Represents the competitive interplay mode. This pattern consists of two RBPs (RBP1 and RBP2), contending for binding to one or more overlapping binding sites on an RNA species. This competition results in a balance of RBP1 and RBP2 bound to these molecules, which determines the outcome of the regulation. **(C)** Describes the mutual interplay mode. Here, two RBPs (RBP1 and RBP2) can control the expression of one another to constrain and fine-tune the outcome of the regulation of their target RNAs. This mechanism can be heterogeneous (up), with RBP2 binding to RBP1 mRNA (or vice-versa) or autogenous (down), where an RBP binds to its cognate mRNA.

The interplay of two RNA-binding proteins having a common regulatory goal, and whose action is synergistic, is said to be *cooperative*. Occurrences of this phenomenon have been observed at the various steps of post-transcriptional regulation and can be mediated by the physical interaction of the RBPs or by them sharing targets without direct contact, as shown in Figure 1A.

**RNA processing** has been observed to rely on RBP cooperation at its various stages. The heterogeneous nuclear ribonucleoproteins (hnRNPs) are a large family of multifunctional RBPs, mainly involved in alternative splicing. HNRNPL, a member of this family, cooperates with PTBP1 to exclude exon P3A of CHRNA1 via a protein-protein interaction mediating HNRNPL binding to the region upstream of that exon in the pre-mRNA (Rahman et al., 2013). Genome-wide analyses found HNRNPM, C, and H as part of a larger RBP complex also including RBFOX2, MATR3, and other proteins. HNRNPM binding sites are enriched in the proximity of RBFOX2 ones, and RBFOX2 was shown to stimulate HNRNPM-mediated splicing repression, suggesting a widespread cooperation of the two RBPs (Damianov et al., 2016). MATR3 instead represses splicing by interacting with PTBP1 (Coelho et al., 2015). Eventually, hnRNPs homodimers were also observed. Multiple HNRNPA1 proteins bind cooperatively by spreading on target transcripts to unwind the RNA secondary structure and allow splicing to occur (Okunola and Krainer, 2009). Another important group of splicing regulators is the serine/arginine (SR) proteins. These RBPs control their targets combinatorially through distinct and overlapping binding sites mostly in a synergistic fashion (Bradley et al., 2015). Also nuclear export and alternative polyadenylation exploit RBP cooperation. ZNF385A (Hzf) and ELAVL1 (HuR) co-regulate p53 export in an RNA-dependent fashion (Nakamura et al., 2011). MSI1 remodels the RNA structure to guide the polyadenylation site choice by CPEB1 in Xenopus (Weill et al., 2017), while PABPC1 recruits HNRNPLL to the 3′end of immunoglobulin mRNA to induce isoform switching through alternative polyadenylation (Peng et al., 2017).

The control of **messenger RNA stability** is another process in which cooperation between RBPs plays an important role. Among the cis-elements involved, AU-rich elements (AREs) are the best-characterized, and several studies uncovered ARE-mediated interplay occurrences. HNRNPF is a required cofactor, through an RNA-independent interaction, for mRNA decay induced by two known ARE-binding proteins (AREBPs), ZFP36, and BRF1 (Reznik et al., 2014). Another AREBP, AUF1, promotes mRNA silencing by binding near AGO2 sites and contributing to its loading with miRNAs (Wu et al., 2013; Min et al., 2017). Genome-wide analyses using RBP binding and gene expression data have also found TIA1 and ELAVL1 to cooperate through AREs in a distance-dependent way. TIA1 and PUM1/2 destabilize their target transcripts, while MSI1 and ELAVL1 stabilize theirs (HafezQorani et al., 2016). ELAVL1 is a well-known regulator of mRNA stability, and further works have shown synergism with other RBPs such as RBM38, which increases ELAVL1 binding through physical interaction at ARE sites (Cho et al., 2010). Also ADAR1, a key player of A-to-I RNA editing, forms RNA-dependent complexes with ELAVL1. The complex controls the degradation rate of ADAR1 editing targets, thus coupling the two processes by a cooperative mechanism (Wang et al., 2013). ADAR1 also coordinates with ADAR2 to modulate editing and stability of the Ctn nuclear RNA through an RNA-dependent interaction, showing that RBP interplay is not limited to mRNAs (Anantharaman et al., 2017a). Other cis-elements also use such control patterns. Two examples are the stability-promoting interaction of LARP4 with PABPC1 (Yang et al., 2010) and the collaborative control of c-MYC mRNA stabilization by IGF2BP1 and HNRNPU, SYNCRIP, YBX1, or DHX9 (Weidensdorfer et al., 2009).

**Figure 2:**
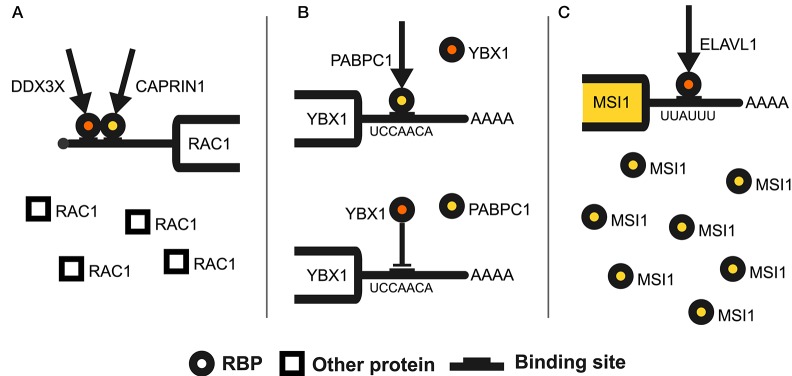
Examples of RBP interplay mechanisms. The figure illustrates an occurrence of each RBP interaction mode. **(A)** Shows the 5′UTR-mediated cooperation of DDX3X and CAPRIN1 which controls the translation of RAC1 mRNA. This RNA-dependent association exploits a further interaction with PABPC1 at the leading edge of the cell to promote fibroblasts migration and spreading (Copsey et al., 2017). **(B)** Depicts the antagonistic interaction of YBX1 and PABPC1 on the 3′UTR of YBX1 mRNA. This two RBPs target overlapping binding sites within the same regulatory element (the YBX1 binding sequence is shown). While PABPC1 stimulates YBX1 mRNA translation in a poly(A)-tail-independent manner, YBX1 attempts to repress it by inhibiting translation initiation (Lyabin et al., 2011). **(C)** Portrays the mutual heterogeneous interaction between the ELAVL1 and MSI1 RBPs. ELAVL1 binds to an AU-rich element in the distal part of the 3′UTR of MSI1 mRNA (the ELAVL1 binding sequence is shown). This binding induces higher steady-state levels of MSI1 mRNA by exerting a stabilizing effect. In turn, this enhances MSI1 translation, ultimately leading to upregulation of its protein levels (Vo et al., 2012).

The impact of cooperative RBP interactions eventually reaches out to **translational control**, as suggested by some recent works. A complex mechanism sees HNRNPA0, HNRNPA2B1, and ELAVL1 concurrently binding to the 5′UTR of ANXA2R mRNA. They focus the translation on its first uORF, thus tightly inhibiting the synthesis of the downstream coding sequence (Zhang et al., 2016). Another 5′UTR-mediated RNA-dependent interaction occurs between DDX3X and CAPRIN1. These RBPs exploit structured UTRs to control translation through PABPC1 and promote cell migration and spreading (Figure 2A) (Copsey et al., 2017). Interactions in translational control also occur within the 3′UTR. The synthesis of selenoproteins relies on the direct interaction between SECISBP2 and the SMN complex. This interaction allows to assemble and translate the selenoprotein mRNP through the selenocysteine insertion sequence element, found in the 3′UTR of these mRNAs (Gribling-Burrer et al., 2017). ZFP36 and TTP mediate a further role of 3′UTR ARE elements in translational control. Indeed, the interaction between ZFP36 and PABPC1 leads to translational repression of ZFP36 target mRNAs in the inflammatory response (Zhang et al., 2017), while ZFP36 and DDX6 inhibit the translation of target mRNAs by shifting them to lighter polysomes (Qi et al., 2012). Eventually, the cooperation of SYNCRIP and MSI2 is critical for myeloid leukemia cells survival, with the two RBPs physically interacting to promote translation of their targets (Vu et al., 2017).

## Competitive target regulation

The interplay of two RNA-binding proteins having different regulatory goals, and whose action is antagonistic, is called *competitive*. This pattern, seen in action at the various steps of post-transcriptional control, involves the formation of a balance of the competing RBPs on the target RNA species, which eventually defines the outcome of the regulation (Figure 1B).

**RNA processing** offers several examples of competitive patterns, mainly involving alternative splicing regulation by the hnRNPs family. Within this family, HNRNPL and HNRNPLL antagonistically modulate the splicing of CHRNA1, with HNRNPL supporting the exclusion of exon P3A and HNRNPLL favoring its inclusion (Rahman et al., 2013). hnRNPs also compete with the other major family of splicing regulators, the SR proteins. In particular SRSF1 blocks cooperative HNRNPA1 binding on exonic splicing silencers. As SRSF2 is unable to do so, exon inclusion levels are defined by the balance of the three RBPs (Zhu et al., 2001). HNRNPC instead prevents the exonization of Alu elements, and the resulting disruption of transcript function, by blocking U2AF2 from binding at cryptic splice sites found in Alu-containing exons (Zarnack et al., 2013). Outside the hnRNP and SR families, genome-wide analyses found a conserved functional antagonism in splicing regulation between CELF2 and RBFOX2. These RBPs bind to overlapping sites on several pre-mRNAs with opposing consequences on exons inclusion (Gazzara et al., 2017). This mechanism is also used by QKI and PTBP1, competing for the splicing of specific exons on almost 200 common targets (Hall et al., 2013). Aside from splicing, other processing steps are also affected by RBP competition. TARDBP (TDP43) and FUS are functionally redundant on HDAC6 mRNA, as they compete for overlapping binding sites but produce the same effect, i.e., tuning its processing and nuclear export (Kim et al., 2010). Eventually, also viral RNA can be controlled by RBP competition. ELAVL1 was indeed shown to compete with PTBP1 to facilitate SSB (La) binding on the 3′UTR of hepatitis C RNA, thus enhancing its replication (Shwetha et al., 2015).

The evidence for antagonistic control of **messenger RNA stability** by RBPs is diverse and, as for cooperative patterns, involves AU-rich elements (AREs) and other mechanisms. Suggesting an ancient origin for RBP competition patterns, Puf proteins were found to form a competitive network of interactions in yeast, regulating target mRNAs decay and translational repression (Lapointe et al., 2017). In human cells, AUF1 and ELAVL1 compete for binding to CDKN1A and CCND1 mRNAs by targeting overlapping sites with opposing effects on mRNA stability (enhancing for ELAVL1 and decay-promoting for AUF1; Lal et al., 2004). ELAVL1 competes likewise with TIA1 for binding to the 3′UTR of PDCD4 mRNA, with the RBPs being functionally redundant as they both induce mRNA stabilization (Wigington et al., 2015). Similarly, ELAVL1 and ZFP36 are broad antagonistic regulators of stability. They bind to overlapping sites, with ZFP36 acting as a destabilizing factor (Mukherjee et al., 2014). AREBPs also interact with other RBPs, as shown by ADAR2 modifying access of ELAVL1 and PARN to Ctn RNA to enhance its stability (Anantharaman et al., 2017b). Globally, 3′UTR regions containing AREs have been theorized as “hotspots” for multiple RBPs binding overlapping sites, fostering competition between these factors and influencing target mRNA stability (Plass et al., 2017). Accordingly, the degradation of most mRNAs starts with deadenylation. Two PAN3 isoforms, PAN3S and PAN3L, compete for PABPC1 to regulate deadenylation with opposite effects. Their interaction sees PAN3S enhancing deadenylation and PAN3L repressing it (Chen et al., 2017).

Eventually, several works have shown occurrences of **translational control** mediated by RBP competition, with translation initiation as the most targeted step. LARP1 competes with eIF4E to control terminal oligopyrimidine (TOP) mRNAs translation. LARP1 binds their cap and adjacent 5′TOP motif, impeding access of eIF4E to the cap and thus blocking eIF4F assembly (Hopkins et al., 2016). MSI1 can instead inhibit the translation of its target mRNAs by competing with eIF4G for PABPC1. MSI1 thus blocks the 80S assembly and leads to stress granules recruitment of stalled preinitiation complexes (Kawahara et al., 2008). Another mechanism involving PABPC1 sees it contending with YBX1 for binding to a regulatory element in YBX1 3′UTR. While PABPC1 stimulates YBX1 translation, YBX1 itself seeks to repress it (Figure 2B) (Lyabin et al., 2011). Also ARE-binding proteins are antagonistic regulators of translation. CELF1 and ELAVL1 compete for a 3′UTR element in MYC mRNA. CELF1 represses MYC translation, without affecting its mRNA levels, by decreasing ELAVL1 association with the mRNA (Liu et al., 2015). CELF1 and ELAVL1 also bind to the same 3′UTR element on OCLN mRNA. CELF1 represses OCLN translation through mRNA translocation to P-bodies while ELAVL1 enhances it by displacing CELF1 (Yu et al., 2013). TIA1 and ELAVL1 instead antagonize for cytochrome c mRNA translation by concurrently binding distinct 3′UTR sites (Kawai et al., 2006).

## Mutual RBP regulation

The RBP interaction pattern in which one regulates the expression of the other is said to be *mutual*. The two RNA-binding proteins can be different (*heterogeneous* interplay), or a single RBP can control its cognate mRNA, a mechanism called *autogenous* regulation (Figure 1C). Accumulating evidence suggests the occurrence of these patterns throughout post-transcriptional regulation. Autogenous regulation, in particular, is increasingly credited to be a general behavior. While it is not competitive or cooperative *per se*, since a single RBP is involved, autogenous regulation is fundamental in shaping RBP interaction patterns and is thus discussed here.

The **heterogeneous interactions** observed so far hint at the presence of an extended RBP regulatory hierarchy, realizing the *regulator-of-regulators* concept (Keene, 2007). This is exemplified by our discovery of a network of 23 RBPs hierarchically controlled by ELAVL1 (Dassi et al., 2013), and by two large-scale analyses of RBP interactions, although including only a fraction of all RBPs (Mittal et al., 2011; Dassi et al., 2016). Further works on ELAVL1 have shown it promoting the stability and translation of MSI1 mRNA (Figure 2C) (Vo et al., 2012). ELAVL1 is itself regulated via alternative polyadenylation by the neuronal ELAV RBPs during neuronal differentiation (Mansfield and Keene, 2012). Splicing is one widely used means for RBPs to control other RBPs expression. RBFOX2 regulates 70 RBPs by modulating alternative splicing-coupled nonsense-mediated decay (AS-NMD) of their mRNA. As these RBPs are frequently under autogenous regulation, RBFOX2 represent a global controller of such behavior (Jangi et al., 2014). The stability of RBFOX2 mRNA is, in turn, decreased by CELF2 to tune the outcome of their splicing antagonism (Gazzara et al., 2017). AS-NMD is also used by RBM10 to repress RBM5 mRNA (Sun et al., 2017), with RBM5 in turn controlling the expression of one splicing variant of RBM10 to reduce its pro-oncogenic role (Loiselle et al., 2017). Eventually, these heterogeneous interactions produce global effects on cell phenotypes. This is shown by the destabilization of EIF4EBP2 mRNA by IGF2BP3, which positively regulates eIF4E to promote translational activation and enhance proliferation of lung adenocarcinoma cells (Mizutani et al., 2016).

**Autogenous interactions** have been observed for at least 57 human RBPs (as per the AURA2 database; Dassi et al., 2014), and are thus increasingly considered to be a mechanism of general significance. Analyzing the human proteome with the catRAPID algorithm highlighted an enrichment of autogenous associations within aggregation-prone disordered proteins (Zanzoni et al., 2013). This suggests autoregulation as a mean to reduce protein expression and prevent the accumulation of toxic aggregates. An example of this phenomenon is TARDBP, which represses its mRNA by binding to a 3′UTR element (Ayala et al., 2011). Autogenous regulation occurs throughout post-transcriptional processes. RBM10 negatively autoregulates its mRNA by promoting AS-NMD (Sun et al., 2017), as also suggested for one RBM10 isoform by a second work (Loiselle et al., 2017). Similar AS-NMD patterns are also employed by FUS (Zhou et al., 2013), PTBP1 (Wollerton et al., 2004), HNRNPA2B1 (McGlincy et al., 2010), HNRNPL (Rossbach et al., 2009), TIA1 and TIAL1 (Le Guiner et al., 2001), SRSF2 (Sureau et al., 2001), and TRA2B (Stoilov, 2004). QKI isoforms control nuclear RNA stability, splicing, and translation to cross-regulate themselves and fine-tune the isoform balance of this key developmental regulator (Fagg et al., 2017). ADAR edits its mRNA to alter localization and reduce its efficiency (Drosophila) or protein levels (Mouse) (Savva et al., 2012). Alternative polyadenylation is targeted by ELAVL1 to produce a longer 3′UTR including a destabilizing element (Dai et al., 2012). Similarly, CSTF3 can use an intronic poly(A) site to attenuate its expression (Luo et al., 2013). An unusual mechanism sees DGCR8 guiding the Microprocessor complex to cleave an hairpin in its 5′UTR, thus negatively regulating its expression (Triboulet et al., 2009). Eventually, YBX1 and PABPC1 autoinhibit their translation initiation by respectively targeting the 3′UTR (Lyabin et al., 2011) or the 5′UTR (Kini et al., 2015).

## Perspectives

Regulatory interactions between RNA-binding proteins occur, in their various forms, throughout all processes composing post-transcriptional control of gene expression. The pervasiveness of these mechanisms suggests that they represent an important additional layer of regulation, providing precision and flexibility to PTR networks.

But where do we stand in their discovery and characterization? As seen in the previous sections, most evidence derives from low-throughput assays, rather than by large-scale analyses of the interacting potential of two RBPs. Our current toolbox is the limiting factor. CLIPs (Wheeler et al., 2017) can only analyze single RBPs in isolation, and few RBPs have been assayed by CLIP yet. SEQRS instead analyses the sequence specificity of RBP complexes by *in vitro* selection and high-throughput sequencing (Campbell et al., 2012). However, being *in vitro* implies it ignores the effect of other factors on mRNA accessibility. Eventually, Loregic (Wang et al., 2015) computationally characterizes the cooperative logic of regulatory factors, requiring both binding and gene expression data. So, to allow large-scale analyses to become commonplace, future experimental work should focus on developing methods to analyze pairs of RBPs concurrently, *in vivo*, and at a genome-wide scale. Further efforts should also include computational tools detecting RBP interactions from binding data, even when suitable gene expression data is unavailable or does not allow detecting such interactions.

The evidence described above suggests that the healthy cell requires finely balanced RBP interactions. Even slight alterations to RBPs, impacting their binding specificity and interaction potential, could indeed favor the development of diseases such as cancer. This makes RBP interplay patterns an attractive target for the design of innovative therapeutic options. However, we know little about how these mechanisms contribute to complex diseases onset and progression, and more efforts should be made toward this goal. In this regard, describing the evolutionary paths giving rise to RBP interactions would also be helpful to understand their role and how they could be exploited to our advantage. Eventually, progressing toward the full characterization of the RBP regulatory hierarchy is paramount. Understanding its architecture will provide us with a dynamic new tool to modulate gene expression.

In conclusion, RBP interactions provide precision, as they fine-tune the expression of their targets through a balanced interplay of regulators. They expand PTR networks, providing the flexibility to evolve complex behaviors by novel combinations of a powerful set of building blocks, the RNA-binding proteins.

## Author contributions

The author confirms being the sole contributor of this work and approved it for publication.

### Conflict of interest statement

The author declares that the research was conducted in the absence of any commercial or financial relationships that could be construed as a potential conflict of interest.

## Abbreviations

RBP: RNA-binding protein
PTR: post-transcriptional regulation of gene expression
PTRN: post-transcriptional regulatory network
ADAR: adenosine deaminase, RNA specific
ADARB1: adenosine deaminase, RNA specific B1
AGO2: argonaute 2, RISC catalytic component
ANXA2R: annexin A2 receptor
BRF1: RNA polymerase III transcription initiation factor subunit
CAPRIN1: cell cycle associated protein 1
CCND1: cyclin D1
CDKN1A: cyclin dependent kinase inhibitor 1
CELF1/2: CUGBP Elav-like family member 1/2
CHRNA1: cholinergic receptor nicotinic alpha 1 subunit
CSTF3: cleavage stimulation factor subunit 3
CPEB1: cytoplasmic polyadenylation element binding protein 1
DDX3X: DEAD-box helicase 3, X-linked
DDX6: DEAD-box helicase 6
DHX9: DExH-box helicase 9
EIF4EBP2: eukaryotic translation initiation factor 4E binding protein 2
ELAVL1: ELAV like RNA binding protein 1
FUS: FUS RNA binding protein
HDAC6: histone deacetylase 6
HNRNPA0,A1,A2B1,C,D,F,L,LL,M,U: heterogeneous nuclear ribonucleoproteinA0,A1,A2B1,C,D,F,L,LL,M,U
IGF2BP1/3: insulin like growth factor 2 mRNA binding protein 1/3
LARP4: La ribonucleoprotein domain family member 4
MATR3: matrin 3
MSI1/2: musashi RNA binding protein 1/2
MYC,: MYC proto-oncogene, bHLH transcription factor
OCLN: occludin
PABPC1: poly(A) binding protein cytoplasmic 1
PAN3: PAN3 poly(A) specific ribonuclease subunit
PARN: poly(A)-specific ribonuclease
PDCD4: programmed cell death 4
PTBP1: polypyrimidine tract binding protein 1
PUM1/2: pumilio RNA binding family member 1/2
QKI, QKI: KH domain containing RNA binding
RBFOX2: RNA binding protein, fox-1 homolog 2
RBM5/10/38: RNA binding motif protein 5/10/38
SECISBP2: SECIS binding protein 2
SMN1: survival of motor neuron 1, telomeric
SRSF1/2: serine and arginine rich splicing factor 1/2
SSB: sjogren syndrome antigen B
SYNCRIP: synaptotagmin binding cytoplasmic RNA interacting protein
TARDBP: TAR DNA binding protein
TIA1: TIA1 cytotoxic granule associated RNA binding protein
TIAL1: TIA1 cytotoxic granule associated RNA binding protein like 1
TRA2B: transformer 2 beta homolog
U2AF2: U2 small nuclear RNA auxiliary factor 2
YBX1: Y-box binding protein 1
ZFP36: ZFP36 ring finger protein
ZNF385A: zinc finger protein 385A.
